# Recent advances in medicinal compounds related to corneal crosslinking

**DOI:** 10.3389/fphar.2023.1232591

**Published:** 2023-09-29

**Authors:** Danyi Qin, Yi Han, Lixiang Wang, Hongbo Yin

**Affiliations:** ^1^ Department of Ophthalmology, West China Hospital, Sichuan University, Chengdu, Sichuan, China; ^2^ Fujian Provincial Key Laboratory of Ophthalmology and Visual Science, Eye Institute and Affiliated Xiamen Eye Center, School of Medicine, Xiamen University, Xiamen, Fujian, China

**Keywords:** corneal crosslinking, medicinal compounds, treatment, agents, enhancers, nanomaterials, supplements

## Abstract

Corneal crosslinking (CXL) is the recognized technique to strengthen corneal collagen fibers through photodynamic reaction, aiming to halt progressive and irregular changes in corneal shape. CXL has greatly changed the treatment for keratoconus (KCN) since it was introduced in the late 1990’s. Numerous improvements of CXL have been made during its developing course of more than 20 years. CXL involves quite a lot of materials, including crosslinking agents, enhancers, and supplements. A general summary of existing common crosslinking agents, enhancers, and supplements helps give a more comprehensive picture of CXL. Either innovative use of existing materials or research and development of new materials will further improve the safety, effectiveness, stability, and general applicability of CXL, and finally benefit the patients.

## 1 Introduction

Collagen crosslinking refers to a method to increase collagen strength through chemical formation of covalent bonds between polypeptide chains of collagen molecules, or between collagen molecules and collagen fibrils. The benefits of collagen crosslinking have been well documented in industry, bioengineering, and stomatology (Hardan et al., 2022; Wang et al., 2023). In ophthalmology, corneal crosslinking (CXL) is mainly used in the treatment of dilated corneal diseases, such as keratoconus (KCN), pellucid marginal corneal degeneration (PMD), and iatrogenic keratectasia, in which KCN has been the most common by far.

Cornea is the protective outer layer of the eye, which not only serves as a barrier against external objects but also the main contributor to total refractive power. The peripheral part of the cornea is the thickest, and it becomes thinner toward its center. Microstructurally, the cornea is organized in five layers, which from superficial to deep are corneal epithelium, Bowman’s membrane, corneal stroma, Descemet’s membrane, and corneal endothelium. The epithelial tight junctions constitute the principle barrier to outside substances to cross the corneal epithelium through the paracellular pathway in a regulated way (Leong and Tong, 2015). The regular packing of corneal collagen with a proteoglycan-rich matrix helps to maintain the corneal transparency, and the bonds between the polymeric chains of proteins serve to increase corneal strength and its resistance against mechanical degradation and deformation (Vohra et al., 2023).

**FIGURE 1:**
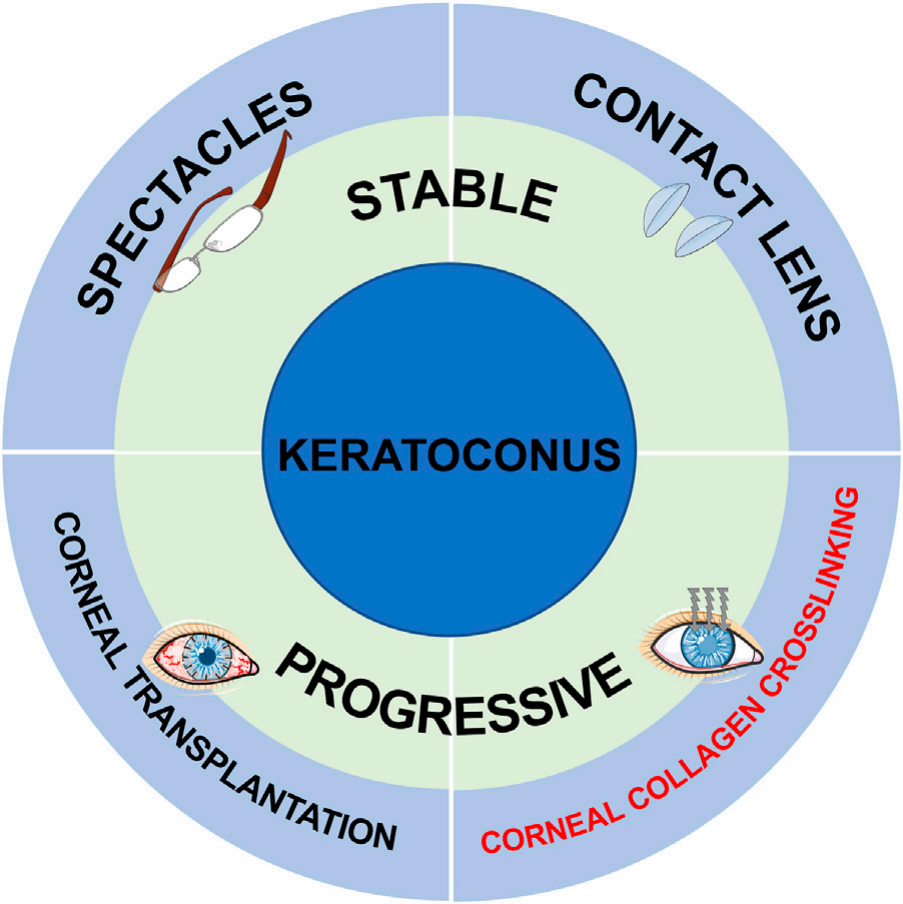
The current treatment strategies for stable and progressive KCN patients. (The Figure was partly generated using Servier Medical Art, provided by Servier, licensed under a Creative Commons Attribution 3.0 unported license).

Corneal ectasia refers to a process of progressive corneal thinning with stromal collagen matrix alterations, leading to irregular protrusion of the cornea and further visual loss. KCN is a congenital developmental abnormality characterized by localized corneal curvature elevation and corneal stroma thinning. KCN usually occurs during adolescence with heterogeneous progression rate, and stablizes in middle age. It is estimated that the prevalence and incidence rates of KCN are 0.2-4790/100000 and 1.5-25/100000 persons per year, respectively (Santodomingo-Rubido et al., 2022). Environmental and genetic factors are known as the major risk factors (Ferrari and Rama, 2020), but the specific pathogenesis of KCN remains poorly understood. Continuous development and improvement of therapeutic strategies have been made. In early stages of KCN, patients are suggested to wear rigid gas permeable (RGP) lenses to control the progression of the disease, while they have to receive corneal transplantation in the advanced stages. Hopefully, the advent of CXL provides progressive KCN patients a novel therapeutic approach to effectively impede the development of the disease (Figure 1).

Wollensak et al. first reported the clinical efficacy of riboflavin-ultraviolet A (RF-UVA) CXL in keratoconus patients in 2003, which was proved efficient to reduce corneal curvature, prevent disease progression, and improve visual acuity (Wollensak et al., 2003). RF-UVA CXL was proved to be safe and effective in stabilizing progressive KCN or corneal ectasia within 12 months in the US multicenter trials (Greenstein and Hersh, 2021). The conventional RF-CXL takes more than 30 min, which is likely to cause corneal dehydration, intraoperative thinning, and consequent infectious complications (Evangelista and Hatch, 2018; Agarwal et al., 2022). To mitigate those risks, accelerated irradiation protocols (A-CXL) with shorter treatment time and higher UVA intensities has been developed. Additionally, high oxygen consumption and subsequent corneal stromal hypoxia caused by continuous UVA illumination might affect crosslinking efficacy. Pulsed A-CXL has therefore been developed to replenish oxygen by withdrawing UVA illumination at intervals. Yet more evidence is needed for their long-term efficacy and stability.

**FIGURE 2:**
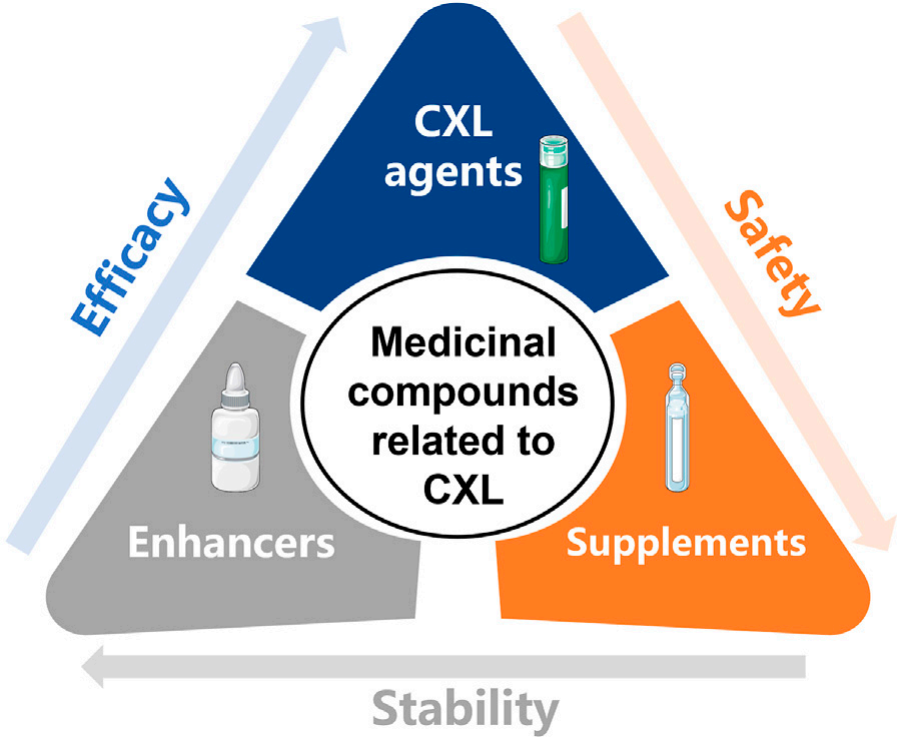
The medicinal compounds related to CXL, including CXL agents, enhancers, and supplements. (The Figure was partly generated using Servier Medical Art, provided by Servier, licensed under a Creative Commons Attribution 3.0 unported license).

Significantly, epithelial debridement is required in conventional RF-CXL to improve the corneal penetrability of riboflavin, which increases the incidence of some major complications, including postoperative pain, delayed corneal epithelial healing, infectious keratitis, corneal opacity, and abnormal wound-healing response. Under the condition of corneal dehydration and corneal thinning, epithelium-off (epi-off) CXL also increases the risks of corneal endothelium, lens, and retina injuries caused by UVA light (Evangelista and Hatch, 2018; Agarwal et al., 2022). To prevent the complications above, great efforts have been made to minimize the corneal exposure to UVA light and avoid corneal epithelial removal. Hence, multifarious strategies to enhance the corneal penetrability of crosslinking agents in epithelium-on (epi-on) CXL and various corneal crosslinking agents other than riboflavin have been developed. Intriguingly, most measures involve the application of novel materials (Figure 2). In this case, here we aim to summarize existing common crosslinking agents, enhancers, and supplements, and demonstrate their characteristics, advantages, and limitations, trying to provide a reference for the development of promising materials in improving CXL efficacy and safety.

## 2 CXL agents

Photosensitizer is the key element in the procedure of CXL, and riboflavin is the most widely used one. Spoerl et al. first described RF-UVA CXL on porcine cornea in 1998, and they pointed that RF-UVA CXL could stiffen the stromal collagen fibers (Spoerl et al., 1998). Then Wollensak et al. reported the clinical efficacy of RF-UVA CXL in keratoconus patients in 2003, and they indicated that RF-UVA CXL could reduce corneal curvature, prevent disease progression, and improve visual acuity (Wollensak et al., 2003). The clinical value of this novel therapeutic technique has been confirmed by numerous clinical trials (Raiskup et al., 2015; Hersh et al., 2017). Meanwhile, many other photosensitizers have been extensively studied, providing a variety of alternatives to riboflavin. The advantages and limitations of the CXL agents described below are summarized in Table 1.

**TABLE 1:** The advantages and limitations of existing CXL agents.

CXL agent	Advantages	Limitations	Mechanisms	References
Riboflavin	Effectiveness and stability	Corneal epithelial removal and subsequent complications	Generating ROS to form covalent bonds between collagen molecules or fibrils	Spoerl et al. (1998), Wollensak et al. (2003), Raiskup et al. (2015), Hersh et al. (2017), Evangelista and Hatch (2018), Agarwal et al. (2022)
Hypo-osmolar riboflavin	Suitable for thin corneas less than 400 μm	Corneal epithelial removal and subsequent complications	Thickening corneas to protect corneal endothelium and intraocular tissues	Nassaralla et al. (2013), Koc et al. (2016), Wollensak and Sporl (2019), Akkaya et al. (2020), Singh et al. (2020), Yurttaser Ocak and Mangan (2021), Celik Buyuktepe and Ucakhan (2022), Salimi et al. (2022)
Genipin	Rapidness and strong operability	Corneal epithelial removal and subsequent complications	Generating ROS to form covalent bonds between collagen molecules or fibrils	Avila et al. (2012), Dias et al. (2015), Avila et al. (2016), Song et al. (2017), Song et al. (2019), Tang et al. (2019), Song et al. (2021)
FARs & BNAs	Intact corneal epithelium, water solubility and good permeability	Potential toxicity and absence of clinical evidence	Generating ROS to promote DNA-DNA and DNA-protein crosslinking	Paik et al. (2009), Paik et al. (2010), Li et al. (2013), Wen et al. (2013), Li et al. (2014), Babar et al. (2015)
Rose Bengal	Time-efficient, and suitable for thin corneas less than 400 μm	Absence of clinical evidence	Singlet oxygen and electron transfer	Cherfan et al. (2013), Bekesi et al. (2016), Fadlallah et al. (2016), Zhu et al. (2016), Bekesi et al. (2017), Lorenzo-Martin et al. (2018), Wang et al. (2018), Wertheimer et al. (2019), Gao et al. (2022)
WST11	Capable to treat corneas of any thickness with excellent safety and efficacy	Absence of clinical evidence	Generating ROS to form covalent bonds between collagen molecules or fibrils	Brekelmans et al. (2017a), Brekelmans et al. (2017b), Hayes et al. (2020)
Tgases	Effectiveness and safety	Absence of clinical evidence	Inducing new peptide bonds between collagen molecules	Wu et al. (2019), Wu et al. (2020), Wu et al. (2022)
NHS, CS, and EDCI	Intact corneal epithelium and low toxicity	Absence of clinical evidence	Activating carboxylate groups to react with primary amine residues on amino acids by forming either O-acylisourea or N-hydroxysuccinimidyl-ester intermediates	Wang et al. (2017), Haneef et al. (2021)
Ru	Biocompatibility and superior biomechanical results	Only two studies	Covalently crosslinking free tyrosine, histidine, and lysine groups under visible light (400-450 nm)	Thangavel et al. (2019), Gulzar et al. (2022)
Verteporfin	Effectiveness	Only one study	Generating ROS under a non-thermal laser (698 nm) light without the production of heat	Alageel et al. (2018)

### 2.1 Riboflavin

Riboflavin, namely, vitamin B2, being a photoactivated chromophore, is an ideal photosensitizer for its wide absorption spectra. It is well tolerated even with full-body absorption. Riboflavin absorbs energy and turns into excited state when exposed to UVA light. Reactive oxygen species (ROS) generated in this process promotecrosslinking by forming covalent bonds between collagen molecules or fibrils. The light absorbing capacity of riboflavin, meanwhile, reduces the risks of UVA light injuries. Dresden protocol, using 0.1% riboflavin in 20% dextran (402.7 mOsmol/L) and 3 mW/cm2 of UVA for 30 min, has been approved by the United States Food and Drug Administration (FDA) in 2016, which effectively treats anterior 300 μm of the stroma.

Molecular weight being 376.37, it can be difficult for water-soluble riboflavin to penetrate the tight junctions between corneal epithelial cells. To improve the corneal penetrability of riboflavin, the standard surgical procedure, epi-off CXL, requires removal of corneal epithelium. Despite better clinical efficacy, epi-off CXL increases the incidence of the complications resulted from epithelial debridement and decreased corneal thickness (Evangelista and Hatch, 2018; Agarwal et al., 2022).

### 2.2 Hypo-osmolar riboflavin

Generally, the requirement of corneal thickness in original CXL is no less than 400 μm. To expand the application scope, various modifications to the formulation of conventional RF-UVA CXL have been proposed. Hypo-osmolar riboflavin has been used to artificially thicken corneas prior to crosslinking. The corneas can swell up to double its thickness when exposed to the hypo-osmolar riboflavin solution. The swelling effect of hypo-osmolar riboflavin seemed to be short-acting, thus corneal thickness should be monitored throughout CXL procedure (Nassaralla et al., 2013). Despite some evidence supporting potential endothelial cell loss (Yurttaser Ocak and Mangan, 2021), CXL using hypo-osmolar riboflavin solution was proved to halt KCN progression safely and efficiently in thin corneas (Koc et al., 2016; Wollensak and Sporl, 2019; Akkaya et al., 2020; Singh et al., 2020; Salimi et al., 2022). The safety and efficacy of hypo-osmolar riboflavin CXL were similar to those of standard CXL during 3-year follow-up (Celik Buyuktepe and Ucakhan, 2022).

### 2.3 Genipin

Genipin is a bioactive substance extracted from Gardenia jasminoides Ellis fruits, with multiple biological functions including antitumor, anti-inflammation, immunosuppression, and antithrombosis. As a natural crosslinking agent, Genipin inducescrosslinking by reacting with the amino acid chains or proteins. In ophthalmology, Genipin has been used in crosslinking on cornea and sclera (Xue et al., 2018).

Genipin crosslinking (GP-CXL) produced significant crosslinking effect with no toxicity (*ex vivo* rabbit) (Avila et al., 2016), which was equivalent to that of Dresden protocol (Dias et al., 2015). GP-CXL was proved safer than RF-UVA CXL in a 24-h observation, in which the concentration of genipin was 0.2% (Song et al., 2017) (*in vivo* rabbit). Another short-term safety evaluation revealed that GP-CXL could be considered low toxicity when the concentration of genipin was lower than 0.25% (Song et al., 2019) (*ex vivo* rabbit). A relatively long-term investigation indicated that 0.25% genipin enhanced the biomechanical properties of rabbit corneas 14 days after CXL with low toxicity (Tang et al., 2019). Lately, Ali Fadlallah et al. described a novel crosslinking method by immersing 0.25% genipin solution with a vacuum ring for 5 min after epithelial removal. This method possessed the features of safety, rapidness and strong operability, but was greatly affected by the preservation of corneal epithelium. Genipin CXL showed better endothelial safety than RFlavin-UVA CXL from a 2-week observation (Song et al., 2021), but there was no difference in the elevation of intraocular pressure (Avila et al., 2012).

In conclusion, GP-CXL provides an ideal option for thin corneas with keratectasia.

### 2.4 Formaldehyde-releasers (FARs) and aliphatic β-nitroalcohols (BNAs)

FARs are usually used as broad-spectrum preservatives in a wide variety of cosmetic and personal care products for their excellent water solubility, wide pH tolerability, and long-lasting preservative effect. Formaldehyde can rapidly penetrate into cellular compartments and crosslink with DNA and proteins to preserve their structures. As a very common crosslinking agent, formaldehyde is widely used in the production of resins, rubber, etc. Natasha Babar et al. tested the crosslinking efficacy of five FARs on intact cornea. The amount of formaldehyde released from each FAR solution was pH- and concentration-dependent, and the presence of tissue amines from corneas conduces to formaldehyde release. It turned out that diazolidinyl urea (DAU) and sodium hydroxymethylglycinate (SMG) presented significant effectiveness with relatively low toxicity (Babar et al., 2015).

Aliphatic β-nitroalcohols (BNAs) are a class of compounds for use as crosslinking agents. BNAs have good permeability owing to small size and water solubility (Wen et al., 2013), have little impact on light transmission (Paik et al., 2009), and have the function to deliver formaldehyde with lower toxicity than formaldehyde (Li et al., 2013). Compared with mono-nitroalcohols, higher order nitroalcohols (HONAs) showed enhanced efficacy and stability (Paik et al., 2010; Li et al., 2014).

Although promising, more evidence is needed to support the safety and efficacy in applying FARs and BNAs to CXL.

### 2.5 Rose Bengal

Rose Bengal is a well-known diagnostic agent for ocular surface damage. Rose Bengal and green light (RGX) is another alternative to conventional RF-UVA CXL. RGX was previously described to seal wounds in cornea and bond amniotic membrane to ocular surface (Verter et al., 2011; Tsao et al., 2012). Daniel Cherfan et al. firstly applied RGX to rabbit eyes to enhance corneal stiffness (Cherfan et al., 2013). It’s safe, time-efficient, and suitable for thin corneas less than 400 μm (Bekesi et al., 2016; Zhu et al., 2016; Bekesi et al., 2017; Lorenzo-Martin et al., 2018; Wang et al., 2018). RGX increased corneal resistance to digestion by collagenase better than RF-UVA CXL (Fadlallah et al., 2016). The photochemistry of Rose Bengal in cornea may diverge from that of riboflavin since Rose Bengal associates tightly to collagen while riboflavin diffuses freely. Singlet oxygen and electron transfer are two mechanisms of RGX to take effect (Wertheimer et al., 2019). Recently, the inclusion of 1.1% HPMC in the formulation was proved helpful in maintaining central corneal thickness during irradiation without affecting the infiltration of Rose Bengal. The involvement of HPMC further improved the clinical safety and efficacy of RGX (Gao et al., 2022). In all, Rose Bengal provides an attractive approach to stiffen corneas for inhibiting progression of KCN and other ectatic disorders.

### 2.6 WST11

WST-11, also known as padeliporfin, is a vascular-acting photosensitizer consisting of water-soluble, palladium-substituted bacteriochlorophyll derivatives, with potential antineoplastic activity. WST-11 can be activated locally when exposed to low-power laser light, generating ROS.

Arie L Marcovich et al. proved that WST-11 and dextran T-500 (WST-D) treatment upon near infrared (NIR) illumination consistently resulted in significant and prolonged corneal stiffening with minimal side effects. WST-D/NIR would not fundamentally change the distribution pattern of collagen fibrils, which further highlighted its safety (Hayes et al., 2020). Brekelmans et al. (2017a) compressed the irradiation time to 1 min *ex vivo* and 5 min *in vivo* without affecting the stiffening effect, which saved the corneas from prolonged exposure to NIR. WST-D/NIR is capable to treat corneas of any thickness without endangering corneal endothelium or deeper ocular structures. Long-term observation, up to 8 months follow-up, revealed excellent safety and efficacy characteristics of WST-D/NIR (Brekelmans et al., 2017b).

Promisingly, WST-D/NIR can be a safe alternative for patients with advanced corneal thinning, especially for those who are unsuited for RF-UVA CXL.

### 2.7 Transglutaminases (Tgases)

Based on the discovery that the expression levels of transglutaminases (Tgases) would be elevated in human corneal keratocytes treated with RF-UVA (Kopsachilis et al., 2013), Tgases were found to successfully stiffen the cornea by inducing new peptide bonds between collagen molecules, without obvious toxicity to endothelium and keratocytes in the cornea (Wu et al., 2019; Wu et al., 2020). Interestingly, topical application of Transglutaminase 2 inducer, namely, retinol palmitate, could also induce the reinforcement of the cornea (Wu et al., 2022).

### 2.8 N-hydroxysuccinimide (NHS), chondroitin sulfate (CS), and 1-ethyl-3-(3-dimethylaminopropyl) carbodi-imide HCl (EDCI)

NHS, CS, and EDCI have been frequently used in tissue engineering applications. CS-NHS CXL could reinforce the corneal mechanics to nearly the same level of RF-UVA treatment, where functionally modified CS in conjunction with NHS formed crosslinks with collagens and proteoglycans in the cornea. Beneficially, CS-NHS CXL downregulated the expression level of proinflammatory genes and cause no obvious apoptosis in keratocytes (Wang et al., 2017). Likewise, NHS/EDCI CXL showed a similar corneal stiffening effect to 15-min conventional RF-UVA treatment with the advantages of intact corneal epithelium and low toxicity, which resulted from the reaction of carboxylate groups and primary amine residues on amino acids by forming either O-acylisourea or N-hydroxysuccinimidyl-ester intermediates (Haneef et al., 2021). These methods might be of great significance to the patients with more advanced disease and thinner corneas.

### 2.9 Ruthenium (Ru)

Ruthenium (Ru) has been commonly used in tissue engineering applications as an essential component of photoinitiator molecules.

The crosslinking effect of gold nanoparticles conjugated with Ru(II) complexes (NCs) in the presence of persulfate was primarily identified by Thangavel et al. (201). Lately, Ayesha Gulzar et al. have proposed a new CXL method by using Tris (bipyridine) ruthenium (II) ([Ru(bpy)3]2+) and sodium persulfate mixture under visible light (430 nm). This system works by covalently crosslinking free tyrosine, histidine, and lysine group on collagen chains in the corneal stroma. This method advantages in multiple aspects such as higher wavelength light, shorter application time, and increased diffusion. Importantly, the crosslinking effect of this method is superior to that of RF-UVA CXL (Gulzar et al., 2022).

### 2.10 Verteporfin

Photodynamic therapy (PDT) using verteporfin has been used for the treatment of choroidal and corneal neovascularization. Verteporfin with nonthermal laser therapy for 6 sequences has been proved to increase corneal mechanical stiffness and resistance to enzymatic collagenase degradation similar to those induced by standard CXL, in which verteporfin is activated by a non-thermal laser light (698 nm) through a photochemical reaction that generates oxygen-free radicals without the production of heat (Alageel et al., 2018).

## 3 Enhancers to promote the penetration of CXL agents

### 3.1 Chemical enhancers

The most frequently used enhancers to promote the penetration of CXL agents are a series of chemical compounds. Generally, they are readily available and easy to use, but there are also troubles of insufficient effectiveness and potential toxicity. The advantages and limitations of the enhancers described below are summarized in Table 2.

**TABLE 2:** The advantages and limitations of the chemical compounds to promote the penetration of CXL agents.

Chemical enhancer	Advantages	Limitations	Mechanisms	References
BAC	Capable to promote the penetration of riboflavin into the cornea without epithelial removal	Inferior biomechanical effect and potential toxicity	Loosen the tight junctions among corneal epithelial cells	Wollensak and Iomdina (2009), Kissner et al. (2010), Koppen et al. (2012), Raiskup et al. (2012), Stojanovic et al. (2012), Armstrong et al. (2013), Torricelli et al. (2014), Gatzioufas et al. (2016a), Akbar et al. (2017), Heikal et al. (2017), Hill et al. (2020)
Tetracaine	Potentially capable to promote the penetration of riboflavin into the cornea without epithelial removal	Undetermined penetration enhancing effect and potential toxicity	Loosen the tight junctions among corneal epithelial cells	Hayes et al. (2008), Armstrong et al. (2013), Wen et al. (2013), Gatzioufas et al. (2016b), Chen et al. (2016)
Ethanol	Potentially capable to promote the penetration of riboflavin into the cornea without epithelial removal	Insufficient penetration enhancing effect and potential toxicity	Loosen the tight junctions among corneal epithelial cells	Samaras et al. (2009), Ozmen et al. (2014), Bilgihan et al. (2022)
Glutaraldehyde	Capable to promote the penetration of riboflavin into the cornea without epithelial removal	Decreased corneal hysteresis and potential toxicity	Form Schiff bases (-N=C-)	Knox Cartwright et al. (2012), Kim et al. (2014), Labate et al. (2015)
NaI	Capable to promote the penetration of riboflavin into the cornea without epithelial removal	Small sample size and uncertain long-term efficacy and safety	Reduce riboflavin photodegradation	Rubinfeld et al. (2018), Rubinfeld et al. (2021), Epstein et al. (2022), Vaidya et al. (2022)
Calcium chelating agents	Capable to promote the penetration of riboflavin into the cornea without epithelial removal	Uncertain stability especially for pediatric patients	Undermine the integrity of tight junctions by binding with Ca2+ in the corneal epithelium	Alhamad et al. (2012), Caporossi et al. (2012), Filippello et al. (2012), Spadea and Mencucci (2012), Caporossi et al. (2013), Morrison and Khutoryanskiy (2014), Rossi et al. (2015), Morrison et al. (2017), Cifariello et al. (2018)
VE-TPGS	Capable to promote the penetration of riboflavin into the cornea without epithelial removal	Small sample size	Enhance drug permeability as a specific riboflavin transporter	Ostacolo et al. (2013), Caruso et al. (2016), Palazzo et al. (2020), Caruso et al. (2021)
CDs	Capable to promote the penetration of riboflavin into the cornea without epithelial removal	Only two studies on bovine corneas *in vitro*	Encapsulate multiple molecules due to their structure of hydrophilic rim and hydrophobic cavity	Morrison et al. (2013), Conde Penedo et al. (2021)

#### 3.1.1 Benzalkonium chloride (BAC)

BAC is a commonly used preservative in ophthalmic medications. BAC has been used to loosen the tight junctions between corneal epithelial cells so as to enhance the penetration of pharmaceutical agents. Anja Kissner et al. found that the permeability of riboflavin solution containing 0.02% BAC in intact corneas of rabbits was similar to that of standard riboflavin solution in deepithelialized corneas (Kissner et al., 2010). This finding was further proved by Frederik Raiskup et al. and Aleksandar Stojanovic et al. subsequently (Raiskup et al., 2012; Stojanovic et al., 2012). Although transepithelial CXL supplemented with BAC showed less biomechanical effect compared with standard CXL (Wollensak and Iomdina, 2009; Koppen et al., 2012; Gatzioufas et al., 2016a), BAC combined with other chemical enhancers like ethylenediaminetetraacetic acid (EDTA), trometamol (Tris), and hydroxypropyl methylcellulose (HPMC), made transepithelial CXL obtain satisfactory stiffening effect and greater endothelial safety (Torricelli et al., 2014; Akbar et al., 2017; Heikal et al., 2017). The corneal stiffness enhanced by BAC-EDTA CXL was found to be more than twice that of standard Epi-off 2 months after the operation (Armstrong et al., 2013). Moreover, simultaneous optimization of the oxygen environment, riboflavin formulation, and UVA protocol might increase the effects of transepithelial CXL to the greatest extent (Hill et al., 2020).

#### 3.1.2 Tetracaine

Tetracaine was previously used by some clinicians to loosen the epithelial tight junctions before CXL. Clinical efficacy of tetracaine-enhanced epi-on CXL was confirmed in patients with bilateral progressive KCN at 12 months (Chen et al., 2016). Although 1% tetracaine administration seemed to be insufficient to permit the penetration of riboflavin into the corneal stroma of porcine eyes (Hayes et al., 2008), 0.5% tetracaine combined with 0.01% BAC showed significantly increased permeability (Wen et al., 2013). There were concerns that tetracaine might cause some severe toxic effects (Gatzioufas et al., 2016b), yet negligible stromal cell death was found tetracaine transepithelial CXL according to Armstrong et al. (2013). Anyhow, sufficient evidence supporting the short-term and long-term safety of tetracaine is needed for its routine clinical use.

#### 3.1.3 Ethanol

Ethanol was used to enhance corneal crosslinking by loosening the tight junctions among corneal epithelial cells. Ethanol application might not be adequate for transepithelial CXL (Samaras et al., 2009; Ozmen et al., 2014), but it achieved satisfactory effectiveness when assisted by iontophoresis, which had a similar effect to standard CXL (Bilgihan et al., 2022).

#### 3.1.4 Glutaraldehyde

Glutaraldehyde is a bifunctional aldehyde, and its two reactive aldehyde groups (-CHO) can react with the amino groups (-NH2) of primary amines nearby to form Schiff bases (-N=C-), which increases the cornea’s mechanical stability. Glutaraldehyde fixation could further increase the stiffness of organ-cultured corneas on the basis of RF-UVA CXL (Knox Cartwright et al., 2012). However, glutaraldehyde also further decreased corneal hysteresis, which reflected the shock-absorbing capacity of corneas (Labate et al., 2015), and it might be highly toxic to normal cells (Kim et al., 2014).

#### 3.1.5 Sodium iodide (NaI)

Rubinfeld et al. (2018) devised a novel transepithelial CXL system, EpiSmart^®^epi-on, which consisted of a new transepithelial riboflavin formulation with a NaI excipient and two sterile proprietary applicators. This system was proved to significantly enhance riboflavin concentration in corneal stroma through intact corneal epithelium. This might be accomplished by enhancing epithelial penetration and reducing riboflavin photodegradation (Rubinfeld et al., 2021). Recently, they have conducted phase 2 trials of EpiSmart^®^epi-on in 1606 KCN patients, and substantiated the excellent safety and efficacy profile of their system during 12-month observation (Epstein et al., 2022; Vaidya et al., 2022).

#### 3.1.6 Calcium chelating agents

Various connexin proteins, such as cadherin, zonula occludens 1 (ZO-1), connexin 43, constitute the tight junctions between corneal epithelial cells with the involvement of calcium ions (Ca^2+^), presenting a physiological barrier for drug delivery (Chu and Zhao, 2020). Calcium chelating agents are supposed to undermine the integrity of tight junctions by binding with Ca^2+^ in the corneal epithelium.

Similar to superficial epithelial scratches, enhanced riboflavin solutions (riboflavin 0.1%, dextran T500 15% with Tris and EDTA) contributed to sufficient riboflavin absorption in corneal stroma (Alhamad et al., 2012). Peter W J Morrison and Vitaliy V Khutoryanskiy investigated the effectiveness of serveral Ca^2+^ sequestering compounds as penetration enhancers in delivery of riboflavin for the treatment of KCN and other corneal disorders, including EDTA, ethylenediamine-N,N′-disuccinic acid (EDDS), and ethylene glycol-bis(2-aminoethylether)-N,N,N′,N′-tetraacetic acid (EGTA). The results revealed that EDTA, EDDS, and EGTA all performed well in reducing electrical resistance of corneal membranes and enhancing corneal permeability of riboflavin. Specifically, EDTA and EGTA brought marked improvement, while EDDS offered moderate enhancement (Morrison and Khutoryanskiy, 2014). Besides laboratory evidence, multiple clinical trials indicated that epi-on CXL with enhanced riboflavin solutions (riboflavin 0.1%, dextran T500 15% with Tris and EDTA sodium salt) had better safety and equivalent clinical outcomes with standard epi-off CXL during up to 2-year follow-ups (Caporossi et al., 2012; Filippello et al., 2012; Rossi et al., 2015; Cifariello et al., 2018). This modified formulation was even safe and effective to ultrathin corneas less than 400 μm during a 1-year follow-up (Spadea and Mencucci, 2012), but there was also scepticism about the stability of its crosslinking effect, especially for pediatric patients under 18 (Caporossi et al., 2013).

Crown ethers are another type of Ca^2+^ sequestering compounds, such as 12-crown-4 (12C4), 15-crown-5 (15C5), and 18-crown-6 (18C6). Crown ethers enhanced the solubility and the penetrability of riboflavin in bovine corneas, smaller-sized of which gave greater enhancement (Morrison et al., 2017).

#### 3.1.7 Vitamin E-tocopherol polyethylene glycol 1000 succinate (VE-TPGS)

Tocopherol polyethylene glycol 1,000 succinate (TPGS) is frequently used in micelles preparation for ocular drug delivery (Ghezzi et al., 2022). VE-TPGS, one of the most common non-ionic surfactants, is effective in enhancing drug permeability through different biological barriers as a specific riboflavin transporter. VE-TPGS together with riboflavin also serves as a topical antioxidant to protect the cornea from ultraviolet damage (Palazzo et al., 2020). Ostacolo et al. (2013) proved that VE-TPGS increased corneal penetration of riboflavin and corneal stiffness in transepithelial CXL on porcine corneas. They further confirmed the effectiveness of VE-TPGS to promote the permeation of riboflavin both in standard and accelerated CXL on KCN patients (Caruso et al., 2021). In a 24-month trial, VE-TPGS contributed to better clinical outcomes in transepithelial CXL with modified UVA irradiation (Caruso et al., 2016). Incidentally, incorporation of riboflavin and VE-TPGS showed a promising effect in crosslinking of dentine in stomatology (Daood et al., 2019; Daood et al., 2020).

#### 3.1.8 Cyclodextrins (CDs)

CDs are a series of water-soluble cyclic oligosaccharides consisting of 6, 7 and 8-(1,4)-linked glucopyranose subunits. The structure of hydrophilic rim and hydrophobic cavity make CDs ideal host molecules. This property enables CDs encapsulate multiple organic and inorganic molecules to form host-guest complexs. CD usually serve as the drug carriers and penetration enhancers in ocular drug delivery (Loftsson and Stefansson, 2017). Peter W J Morrison et al. found that β-cyclodextrin enhanced the solubility and permeability of riboflavin better than α-cyclodextrin. That’s probably because the cavity of β-cyclodextrin (6.0–6.5 Å) was exactly large enough to accommodate a wide range of drugs, while that of α-cyclodextrin (4.7–5.3 Å) was too small to accommodate the aromatic ring of riboflavin (Morrison et al., 2013). Recently, Andrea Conde Penedo et al. discovered that hydroxypropyl-β-cyclodextrin (HPβCD) and sulfobuthylether-β-cyclodextrin (SBEβCD) increased the solubility of riboflavin on bovine corneas. The permeability coefficient was markedly elevated when chitosan and arginine were added to the formulation, and reached the best when riboflavin was combined with 40% (w/v) HPβCD, 0.5% (w/w) arginine, and 0.5% (w/w) chitosan. All the formulations exhibited high safety without apparent side effects (Conde Penedo et al., 2021).

### 3.2 Nanomaterials

The lipophilic property of corneal epithelium makes it an effective barrier to hydrophilic drugs such as riboflavin. A variety of methods have been developed to enhance transepithelial absorption of hydrophilic therapeutic agents, one of which is nanotechnology. Nanomaterials are potent drug delivery vehicles for ophthalmic use due to their sustained effect and enhanced permeability. The amphiphilic components of nanomaterials and the internal migration of drugs contribute to an impressing performance in drug delivery. In addition, the incorporation of nanostructures alone in corneal stroma is supposed to be a promising alternative, as well. The modified corneas implemented with carbon nanostructures showed improved corneal strength (Silvestre et al., 2022). The advantages and limitations of the nanomaterials described below are summarized in Table 3.

**TABLE 3:** The advantages and limitations of the nanomaterials to promote the penetration of CXL agents.

Nanomaterial	Advantages	Limitations	Mechanisms	References
MEs	Potentially capable to promote the penetration of riboflavin into the cornea without epithelial removal	Inferior biomechanical effect	Enhance drug solubility and permeability due to their stability, small size, diverse structures, low surface tension, high loading capacities	Bottos et al. (2013), Lidich et al. (2016), Lidich et al. (2019)
MOFs	Capable to promote the penetration of riboflavin into the cornea without epithelial removal	Only one study on rabbit cornea *in vivo*		Yang et al. (2022)
Nanoparticles	Capable to promote the penetration of riboflavin into the cornea without epithelial removal	Only two studies on donor human sclerocorneal tissues *in vitro*		Labate et al. (2017), Lombardo et al. (2017)
NLCs	Capable to promote the penetration of riboflavin into the cornea without epithelial removal	Only one study on rabbit cornea *in vitro*		Aytekin et al. (2020)

#### 3.2.1 Microemulsions (MEs)

MEs are nano-sized mixtures of water, oil, surfactants, cosurfactants, and electrolytes. Many fascinating properties of MEs, such as thermodynamic stability, small size, and low surface tension, serve to enhance the solubility and permeability of hydrophilic and lipophilic molecules, and improve the sustained and controlled release of applied drugs at the target site (Ustundag Okur et al., 2020).

According to the research of Katia M Bottos et al., in 2013, riboflavin-5-phosphate nanoemulsion achiever greater stromal concentration of riboflavin than standard protocol after 240 min (Bottos et al., 2013). However, the study of Lidich et al. (2019) found that the improvement of biomechanical stiffness using ME loaded with riboflavin phosphate was only nearly a quarter that of standard CXL with epithelial debridement. They previously discovered that the mobility of riboflavin in ME systems was constrained by interactions with the surfactants and cosurfactants, and free transport of drug molecules was achieved in ME containing over 80wt% water (Lidich et al., 2016). Based on this discovery, they prepared riboflavin phosphate-loaded ME, which was diluted up to 97 wt% water content and resulted in final 0.24 wt% riboflavin phosphate, and they applied the ME on rabbit eyes without epithelial debridement. In spite of enhanced permeability of riboflavin in corneal stroma, the increase in biomechanical stiffness of the cornea was not that satisfactory (Lidich et al., 2019). Although it’s not quite clear whether higher stromal concentration of riboflavin and greater crosslinking effect can be achieved with ME systems, these initial attempts offered a potential prospect for utilization of MEs to enhance the penetration of riboflavin in transepithelial CXL.

#### 3.2.2 Nanosized metal–organic frameworks (MOFs)

MOFs are generally acknowledged to be advantageous to act as drug carriers for their special physicochemical features of diverse structures, extended surface areas, high loading capacities, modifiable pore sizes, good biocompatibility, and biodegradability (Yang and Yang, 2020).

Mei Yang et al. developed novel hibiscus-like riboflavin@ZIF-8 nanoflake (6RF@ZIF-8 NF) using zeolitic imidazolate framework-8 (ZIF-8) nanomaterials as carriers. The positive potential of ZIF-8, which neutralized the negative potential of corneal epithelium, and its hydrophobicity facilitated the penetration through the epithelium. The special hibiscus-like structures enlarged the contact area with the epithelium and shortened the release passage of riboflavin. 6RF@ZIF-8 NF CXL demonstrated outstanding transepithelial CXL efficacy, which was slightly better than the conventional CXL, as well as excellent biocompatibility. More importantly, it avoided epithelial debridement and relevant complications (Yang et al., 2022).

#### 3.2.3 Nanoparticles

A new nanotechnology-based platform to deliver riboflavin into the corneal stroma has been built by Strano’s team, which consists of polymeric nanoparticles of 2-hydroxypropyl-β-cyclodextrin, enhancers (EDTA and Tris), and 9-min UVA irradiation with a 10 mW/cm^2^ device. This platform was proved effective in enriching the anterior corneal stroma with riboflavin through the intact epithelium and obtaining obvious stiffening effect, which was better than the effect of conventional CXL (Labate et al., 2017; Lombardo et al., 2017).

#### 3.2.4 Nanostructured lipid carriers (NLCs)

Eren Aytekin et al. loaded NLCs with riboflavin phosphate with permeation enhancer Transcutol P. This system performed well in CXL with high drug loading content, high permeability, and low accumulation in the cornea (Aytekin et al., 2020), which might be a potential alternative for non-invasive KCN treatments.

## 4 Supplements

Supplements such as dextran and HPMC as part of the riboflavin solution can be very helpful in increasing the viscosity of the solution and prolonging the contact time of riboflavin with the cornea. They also help to maintain normal corneal structure and absorb UVA light during CXL procedure. These functions contribute to improve the effectiveness, efficiency and safety of RF-UVA CXL.

### 4.1 Dextran and hydroxypropyl methylcellulose (HPMC)

0.1% Riboflavin 5′-phosphtae in 20% dextran ophthalmic solution has been approved by the United States Food and Drug Administration (FDA). However, dextran-based riboflavin solutions significantly reduced corneal thickness during CXL (Belin et al., 2018), while HPMC-based riboflavin solutions have little impact on it (Zaheer et al., 2018).

Consequently, HPMC has received considerable attention as an alternative substituted for dextran. Compared with dextran riboflavin, HPMC riboflavin might present improved CXL effect resulted from enhanced concentration of riboflavin in the cornea and a deeper demarcation line (Malhotra et al., 2017; Thorsrud et al., 2019). Nevertheless, dextran riboflavin might result in slightly stronger biomechanical properties and better visual outcomes (demonstrated by visual acuity) (Rapuano et al., 2018; Fischinger et al., 2021). As for safety, HPMC riboflavin performed as well as dextran riboflavin (De Paula et al., 2020).

Similarly, dextran and HPMC both exert their effects by maintaining the osmotic pressure of corneal matrix, improving UVA absorption, and increase the viscosity of crosslinking solutions. Even so, more experimental and clinical studies and precisely described protocols are needed for rational use of these supplements. The advantages and limitations of the compounds described above are summarized in Table 4.

**TABLE 4:** The advantages and limitations of the supplements used in CXL.

Supplement	Advantages	Limitations	Mechanisms	References
Dextran	Better biomechanical properties and visual outcomes	Reduced corneal thickness during CXL	1. Maintain the osmotic pressure of corneal matrix; 2. Improve UVA absorption to protect corneal endothelium and intraocular tissues; 3. Increase the viscocity of crosslinking solutions to extend contact time	Belin et al. (2018), Rapuano et al. (2018), De Paula et al. (2020), Fischinger et al. (2021)
HPMC	Better crosslinking effect and little impact on corneal thickness	Uncertain long-term stability		Malhotra et al. (2017), Zaheer et al. (2018), Thorsrud et al. (2019), De Paula et al. (2020)

## 5 Summary and future directions

The corneal stroma makes up approximately 90% of the corneal structure. The collagen fibers and extracellular matrix (ECM) in the corneal stroma are organized into a complex, highly intertwined, 3-dimensional meshwork of transversely oriented fibers, which provides some important properties necessary for functions including transparency, avascularity, physical strength, and maintenance of shape (Ma et al., 2018). Corneal ectasia is a progressive, degenerative ocular disease characterized by corneal bulge, thinning, and structural changes, leading to alterations in refractive power and further vision loss. KCN, PMD, and iatrogenic keratectasia are different subtypes of corneal ectasia, in which KCN is the most common one. Collagen crosslinking refers to a process of forming strong chemical bonds among collagen fibrils to enhance the strength of the material. CXL has now become a recognized technique to strengthen corneal collagen fibers through photodynamic reaction, aiming to halt progressive and irregular changes in corneal shape (Greenstein and Hersh, 2021). CXL involves quite a lot of materials, including crosslinking agents, enhancers, and supplements. A general summary of existing common crosslinking agents, enhancers, and supplements helps give a more comprehensive picture of CXL, and it also helps ophthalmologists and researchers to develop optimal materials for CXL with better efficacy and biosafety.

CXL has greatly changed the treatment for KCN since it was introduced in the late 1990s. For this reason, there has been a significant decline of corneal transplantation on KCN. Numerous improvements of CXL have been made during its developing course of more than 20 years. A-CXL reduces intraoperative and postoperative complications by shortening the operation time. Pulsed A-CXL improves crosslinking efficacy by replenishing oxygen to corneal stroma. To improve the vision quality, a series of CXL-Plus strategies have been proposed, which combine multiple techniques with CXL, such as photorefractive keratectomy (PRK), intracorneal ring segments (ICRS), or phakic intraocular lens implantation (PIOL) (Al-Mohaimeed, 2019). Limited riboflavin penetration through corneal epithelium is a key problem in epi-on CXL. Iontophoresis-assisted CXL increases riboflavin penetration into corneal stroma by applying a small electric current. Furthermore, various infiltration enhancers have been developed, including many chemical substances and nanomaterials. Most chemical substances exert their effect by loosening the tight junctions between corneal epithelial cells, but with certain toxicity, correspondingly. With the rocketing development of nanotechnology, nanomaterials have been applied to deliver riboflavin into the corneal stroma, including polymeric and inorganic nanoparticles. These nano-based drug delivery systems have their own advantages of bioavailability, biocompatibility, high drug loading content and sustained-release effect. Moreover, smart engineering of nanomaterials endows them with multifunctional capabilities, bringing new possibilities in drug delivery, real-time imaging, individualized treatment, and so on (Diez-Pascual and Rahdar, 2022). Combination treatment of different techniques should also be put into consideration in order to achieve better performance. In all, nanotechnology enjoys huge potential and broad prospect in delivering CXL agents efficiently, which can not only improve the performance of agents and improve therapeutic efficacy but also reduce certain side effects and expand the scope of treatment. However, it’s worth noting that the short-term and long-term safety, the complexity of manufacturing, the cost of production, and regulatory restrictions should all be considered. In addition, the vast majority of the discussed materials are still in the laboratory stage, and more translations from bench to bedside are needed.

Ideal crosslinking solutions are better to be formulated to have both maximal ocular contact time as well as corneal permeability, which is more likely to acquire maximum bioavailability. To achieve this, increasing the viscosity of the solution is considered an effective way. Hyperviscosity agents such as dextran and HPMC are commonly used in crosslinking solutions to improve corneal penetration of rivoflavin by increasing tissue contact time. Solutions with the viscosity of up to 20 centipoise (cP) are well tolerated by the eye, increasing tissue bioavailability of the agent. While solutions with the viscosity over 20 cP will result in decreased drug efficacy due to significant irritation and increased tear drainage (Mehta et al., 2020). Hence, to find the balance between maximal drug activity and minimal eye irritation is very important, and this may be where the development of future CXL supplements go for.

Safety, effectiveness and stability are the basic requirements of CXL treatment, and they are interconnected and interacted. To develop CXL-related medicinal compounds, safety is the most fundamental element. Without safety, there is no point of effectiveness and stability. Effectiveness is another essential factor of CXL, which is reflected in corneal curvature reduction, disease progression prevention, visual acuity improvement, and etc. The development of CXL-related medicinal compounds is aimed to achieve better or non-inferior clinical outcomes, compared with existing ones used in clinical. Without effectiveness, it can be difficult to be well accepted by the market and widely used in practice. As for stability, it has further guaranteed the effectiveness and safety of CXL. Therefore, safety, effectiveness and stability are three vital ingredients in the development, production and marketing of CXL-related medicinal compounds. It is important to realize that there is no such substance as a perfectly safe and efficient material for use in CXL. However, this is where true innovations come in. With greater access to the data from basic research and clinical practice, scientists and ophthalmologists will be able to further develop better CXL therapies. There are good reasons to be optimistic about the prospects ahead. Either innovative use of existing materials or research and development of new materials will further improve the safety, effectiveness, stability, and general applicability of CXL, and finally benefit the patients.

## References

[B1] Agarwal R. Jain P. Arora R. (2022). Complications of corneal collagen cross-linking. Indian J. Ophthalmol. 70, 1466–1474. 10.4103/ijo.IJO_1595_21 35502012 PMC9333012

[B2] Akbar B. Intisar-Ul-Haq R. Ishaq M. Arzoo S. Siddique K. (2017). Transepithelial corneal crosslinking in treatment of progressive keratoconus: 12 months' clinical results. Pak J. Med. Sci. 33, 570–575. 10.12669/pjms.333.11907 28811773 PMC5510105

[B3] Akkaya S. Ulusoy D. M. Duru Z. Demirtas A. A. (2020). Long-term outcomes of accelerated corneal cross-linking in the treatment of keratoconus: Comparison of hypotonic riboflavin solution with standard riboflavin solution. J. Refract Surg. 36, 110–117. 10.3928/1081597X-20191218-01 32032432

[B4] Al-Mohaimeed M. M. (2019). Combined corneal CXL and photorefractive keratectomy for treatment of keratoconus: A review. Int. J. Ophthalmol. 12, 1929–1938. 10.18240/ijo.2019.12.16 31850179 PMC6901893

[B5] Alageel S. A. Arafat S. N. Salvador-Culla B. Kolovou P. E. Jahanseir K. Kozak A. (2018). Corneal cross-linking with verteporfin and nonthermal laser therapy. Cornea 37, 362–368. 10.1097/ICO.0000000000001473 29176450 PMC8496940

[B6] Alhamad T. A. O'brart D. P. O'brart N. A. Meek K. M. (2012). Evaluation of transepithelial stromal riboflavin absorption with enhanced riboflavin solution using spectrophotometry. J. Cataract. Refract Surg. 38, 884–889. 10.1016/j.jcrs.2011.11.049 22520311

[B7] Armstrong B. K. Lin M. P. Ford M. R. Santhiago M. R. Singh V. Grossman G. H. (2013). Biological and biomechanical responses to traditional epithelium-off and transepithelial riboflavin-UVA CXL techniques in rabbits. J. Refract Surg. 29, 332–341. 10.3928/1081597X-20130415-04 23659231 PMC6028182

[B8] Avila M. Y. Gerena V. A. Navia J. L. (2012). Corneal crosslinking with genipin, comparison with UV-riboflavin in *ex-vivo* model. Mol. Vis. 18, 1068–1073. 22605919 PMC3351405

[B9] Avila M. Y. Narvaez M. Castaneda J. P. (2016). Effects of genipin corneal crosslinking in rabbit corneas. J. Cataract. Refract Surg. 42, 1073–1077. 10.1016/j.jcrs.2016.04.025 27492108

[B10] Aytekin E. Ozturk N. Vural I. Polat H. K. Cakmak H. B. Calis S. (2020). Design of ocular drug delivery platforms and *in vitro* - *in vivo* evaluation of riboflavin to the cornea by non-interventional (epi-on) technique for keratoconus treatment. J. Control Release 324, 238–249. 10.1016/j.jconrel.2020.05.017 32413453

[B11] Babar N. Kim M. Cao K. Shimizu Y. Kim S. Y. Takaoka A. (2015). Cosmetic preservatives as therapeutic corneal and scleral tissue cross-linking agents. Invest. Ophthalmol. Vis. Sci. 56, 1274–1282. 10.1167/iovs.14-16035 25634979 PMC4338628

[B12] Bekesi N. Gallego-Munoz P. Ibares-Frias L. Perez-Merino P. Martinez-Garcia M. C. Kochevar I. E. (2017). Biomechanical changes after *in vivo* collagen cross-linking with rose bengal-green light and riboflavin-UVA. Invest. Ophthalmol. Vis. Sci. 58, 1612–1620. 10.1167/iovs.17-21475 28297026

[B13] Bekesi N. Kochevar I. E. Marcos S. (2016). Corneal biomechanical response following collagen cross-linking with rose bengal-green light and riboflavin-UVA. Invest. Ophthalmol. Vis. Sci. 57, 992–1001. 10.1167/iovs.15-18689 26968733

[B14] Belin M. W. Lim L. Rajpal R. K. Hafezi F. Gomes J. A. P. Cochener B. (2018). Corneal cross-linking: Current USA status: Report from the cornea society. Cornea 37, 1218–1225. 10.1097/ICO.0000000000001707 30067537

[B15] Bilgihan K. Uysal B. S. Ozmen M. C. Eskalen O. Gurelik G. (2022). Transepithelial diluted alcohol and iontophoresis-assisted corneal crosslinking for progressive keratoconus in adults: 4-Year clinical results. Cornea 41, 462–469. 10.1097/ICO.0000000000002821 34743098

[B16] Bottos K. M. Oliveira A. G. Bersanetti P. A. Nogueira R. F. Lima-Filho A. A. Cardillo J. A. (2013). Corneal absorption of a new riboflavin-nanostructured system for transepithelial collagen cross-linking. PLoS One 8, e66408. 10.1371/journal.pone.0066408 23785497 PMC3681906

[B17] Brekelmans J. Goz A. Dickman M. M. Brandis A. Sui X. Wagner H. D. (2017a). Corneal stiffening by a bacteriochlorophyll derivative with dextran and near-infrared light: Effect of shortening irradiation time up to 1 minute. Cornea 36, 1395–1401. 10.1097/ICO.0000000000001272 28644240

[B18] Brekelmans J. Goz A. Dickman M. M. Brandis A. Sui X. Wagner H. D. (2017b). Long-term biomechanical and histologic results of WST-D/NIR corneal stiffening in rabbits, up to 8 Months follow-up. Invest. Ophthalmol. Vis. Sci. 58, 4089–4095. 10.1167/iovs.17-22108 28828480

[B19] Caporossi A. Mazzotta C. Baiocchi S. Caporossi T. Paradiso A. L. (2012). Transepithelial corneal collagen crosslinking for keratoconus: qualitative investigation by *in vivo* HRT II confocal analysis. Eur. J. Ophthalmol. 22 (7), S81–S88. 10.5301/ejo.5000125 22344471

[B20] Caporossi A. Mazzotta C. Paradiso A. L. Baiocchi S. Marigliani D. Caporossi T. (2013). Transepithelial corneal collagen crosslinking for progressive keratoconus: 24-month clinical results. J. Cataract. Refract Surg. 39, 1157–1163. 10.1016/j.jcrs.2013.03.026 23790530

[B21] Caruso C. Epstein R. L. Troiano P. Napolitano F. Scarinci F. Costagliola C. (2021). Topo-pachimetric accelerated epi-on cross-linking compared to the dresden protocol using riboflavin with vitamin E TPGS: Results of a 2-year randomized study. J. Clin. Med. 10, 3799. 10.3390/jcm10173799 34501248 PMC8432027

[B22] Caruso C. Ostacolo C. Epstein R. L. Barbaro G. Troisi S. Capobianco D. (2016). Transepithelial corneal cross-linking with vitamin E-enhanced riboflavin solution and abbreviated, low-dose UV-A: 24-Month clinical outcomes. Cornea 35, 145–150. 10.1097/ICO.0000000000000699 26606293 PMC4705913

[B23] Celik Buyuktepe T. Ucakhan O. O. (2022). Long-term visual, refractive, tomographic and aberrometric outcomes of corneal collagen crosslinking (CXL) with or without hypoosmolar riboflavin solution in the treatment of progressive keratoconus patients with thin corneas. Graefes Arch. Clin. Exp. Ophthalmol. 260, 1225–1235. 10.1007/s00417-021-05314-w 34837507

[B24] Chen S. Chan T. C. Zhang J. Ding P. Chan J. C. Yu M. C. (2016). Epithelium-on corneal collagen crosslinking for management of advanced keratoconus. J. Cataract. Refract Surg. 42, 738–749. 10.1016/j.jcrs.2016.02.041 27255251

[B25] Cherfan D. Verter E. E. Melki S. Gisel T. E. Doyle F. J. Jr. Scarcelli G. (2013). Collagen cross-linking using rose bengal and green light to increase corneal stiffness. Invest. Ophthalmol. Vis. Sci. 54, 3426–3433. 10.1167/iovs.12-11509 23599326 PMC4597485

[B26] Chu C. C. Zhao S. Z. (2020). Pathophysiological role and drug modulation of calcium transport in ocular surface cells. Curr. Med. Chem. 27, 5078–5091. 10.2174/0929867326666190619114848 31237195

[B27] Cifariello F. Minicucci M. Di Renzo F. Di Taranto D. Coclite G. Zaccaria S. (2018). Epi-off versus epi-on corneal collagen cross-linking in keratoconus patients: A comparative study through 2-year follow-up. J. Ophthalmol. 2018, 4947983. 10.1155/2018/4947983 30151277 PMC6087595

[B28] Conde Penedo A. Diaz Tome V. Fernandez Ferreiro A. Gonzalez Barcia M. Otero Espinar F. J. (2021). Enhancement in corneal permeability of riboflavin using cyclodextrin derivates complexes as a previous step to transepithelial cross-linking. Eur. J. Pharm. Biopharm. 162, 12–22. 10.1016/j.ejpb.2021.02.012 33667681

[B29] Daood U. Matinlinna J. P. Fawzy A. S. (2019). Synergistic effects of VE-TPGS and riboflavin in crosslinking of dentine. Dent. Mater 35, 356–367. 10.1016/j.dental.2018.11.031 30528297

[B30] Daood U. Sauro S. Pichika M. R. Omar H. Liang Lin S. Fawzy A. S. (2020). Novel riboflavin/VE-TPGS modified universal dentine adhesive with superior dentine bond strength and self-crosslinking potential. Dent. Mater 36, 145–156. 10.1016/j.dental.2019.11.003 31818524

[B31] De Paula T. A. A. Cresta F. B. Alves M. R. (2020). Comparative two-photon fluorescence microscopy analysis of riboflavin penetration in two different solutions: Dextran and hydroxypropyl methylcellulose. Clin. Ophthalmol. 14, 1867–1874. 10.2147/OPTH.S258603 32669833 PMC7337433

[B32] Dias J. Diakonis V. F. Lorenzo M. Gonzalez F. Porras K. Douglas S. (2015). Corneal stromal elasticity and viscoelasticity assessed by atomic force microscopy after different cross linking protocols. Exp. Eye Res. 138, 1–5. 10.1016/j.exer.2015.06.015 26093276 PMC4553073

[B33] Diez-Pascual A. M. Rahdar A. (2022). Functional nanomaterials in biomedicine: Current uses and potential applications. ChemMedChem 17, e202200142. 10.1002/cmdc.202200142 35729066 PMC9544115

[B34] Epstein R. J. Belin M. W. Gravemann D. Littner R. Rubinfeld R. S. (2022). EpiSmart crosslinking for keratoconus: A phase 2 study. Cornea 42, 858–866. 10.1097/ICO.0000000000003136 36173242 PMC10234322

[B35] Evangelista C. B. Hatch K. M. (2018). Corneal collagen cross-linking complications. Semin. Ophthalmol. 33, 29–35. 10.1080/08820538.2017.1353809 28876968

[B36] Fadlallah A. Zhu H. Arafat S. Kochevar I. Melki S. Ciolino J. B. (2016). Corneal resistance to keratolysis after collagen crosslinking with rose bengal and green light. Invest. Ophthalmol. Vis. Sci. 57, 6610–6614. 10.1167/iovs.15-18764 27926752

[B37] Ferrari G. Rama P. (2020). The keratoconus enigma: A review with emphasis on pathogenesis. Ocul. Surf. 18, 363–373. 10.1016/j.jtos.2020.03.006 32234342

[B38] Filippello M. Stagni E. O'brart D. (2012). Transepithelial corneal collagen crosslinking: bilateral study. J. Cataract. Refract Surg. 38, 283–291. 10.1016/j.jcrs.2011.08.030 22104644

[B39] Fischinger I. Seiler T. G. Wendelstein J. Tetz K. Fuchs B. Bolz M. (2021). Biomechanical response after corneal cross-linking with riboflavin dissolved in dextran solution versus hydroxypropyl methylcellulose. J. Refract Surg. 37, 631–635. 10.3928/1081597X-20210610-04 34506235

[B40] Gao R. Yan M. Chen M. Hayes S. Meek K. M. He H. (2022). The impact of different rose bengal formulations on corneal thickness and the efficacy of rose bengal/green light corneal cross-linking in the rabbit eye. J. Refract Surg. 38, 450–458. 10.3928/1081597X-20220601-03 35858194

[B41] Gatzioufas Z. Raiskup F. O'brart D. Spoerl E. Panos G. D. Hafezi F. (2016a). Transepithelial corneal cross-linking using an enhanced riboflavin solution. J. Refract Surg. 32, 372–377. 10.3928/1081597X-20160428-02 27304600

[B42] Gatzioufas Z. Sabatino F. Angunawela R. (2016b). Tetracaine-enhanced transepithelial corneal collagen crosslinking. J. Cataract. Refract Surg. 42, 1106. 10.1016/j.jcrs.2016.06.021 27492112

[B43] Ghezzi M. Ferraboschi I. Delledonne A. Pescina S. Padula C. Santi P. (2022). Cyclosporine-loaded micelles for ocular delivery: Investigating the penetration mechanisms. J. Control Release 349, 744–755. 10.1016/j.jconrel.2022.07.019 35901859

[B44] Greenstein S. A. Hersh P. S. (2021). Corneal crosslinking for progressive keratoconus and corneal ectasia: Summary of US multicenter and subgroup clinical trials. Transl. Vis. Sci. Technol. 10, 13. 10.1167/tvst.10.5.13 PMC8740531 34967830

[B45] Gulzar A. Yildiz E. Kaleli H. N. Nazeer M. A. Zibandeh N. Malik A. N. (2022). Ruthenium-induced corneal collagen crosslinking under visible light. Acta Biomater. 147, 198–208. 10.1016/j.actbio.2022.05.040 35643198

[B46] Haneef A. Giridhara Gopalan R. O. Rajendran D. T. Nunes J. Kuppamuthu D. Radhakrishnan N. (2021). Chemical cross-linking of corneal tissue to reduce progression of loss of sight in patients with keratoconus. Transl. Vis. Sci. Technol. 10, 6. 10.1167/tvst.10.5.6 PMC8088226 34003973

[B47] Hardan L. Daood U. Bourgi R. Cuevas-Suarez C. E. Devoto W. Zarow M. (2022). Effect of collagen crosslinkers on dentin bond strength of adhesive systems: A systematic review and meta-analysis. Cells, 11 2417. 10.3390/cells11152417 35954261 PMC9368291

[B48] Hayes S. Aldahlawi N. Marcovich A. L. Brekelmans J. Goz A. Scherz A. (2020). The effect of bacteriochlorophyll derivative WST-D and near infrared light on the molecular and fibrillar architecture of the corneal stroma. Sci. Rep. 10, 9836. 10.1038/s41598-020-66869-y 32555309 PMC7299946

[B49] Hayes S. O'brart D. P. Lamdin L. S. Doutch J. Samaras K. Marshall J. (2008). Effect of complete epithelial debridement before riboflavin-ultraviolet-A corneal collagen crosslinking therapy. J. Cataract. Refract Surg. 34, 657–661. 10.1016/j.jcrs.2008.02.002 18361990

[B50] Heikal M. A. Soliman T. T. Fayed A. Hamed A. M. (2017). Efficacy of transepithelial corneal collagen crosslinking for keratoconus: 12-month follow-up. Clin. Ophthalmol. 11, 767–771. 10.2147/OPTH.S129037 28461739 PMC5408943

[B51] Hersh P. S. Stulting R. D. Muller D. Durrie D. S. Rajpal R. K. United States Crosslinking Study G. (2017). United States multicenter clinical trial of corneal collagen crosslinking for keratoconus treatment. Ophthalmology 124, 1259–1270. 10.1016/j.ophtha.2017.03.052 28495149

[B52] Hill J. Liu C. Deardorff P. Tavakol B. Eddington W. Thompson V. (2020). Optimization of oxygen dynamics, UV-A delivery, and drug formulation for accelerated epi-on corneal crosslinking. Curr. Eye Res. 45, 450–458. 10.1080/02713683.2019.1669663 31532699

[B53] Kim M. Takaoka A. Hoang Q. V. Trokel S. L. Paik D. C. (2014). Pharmacologic alternatives to riboflavin photochemical corneal cross-linking: A comparison study of cell toxicity thresholds. Invest. Ophthalmol. Vis. Sci. 55, 3247–3257. 10.1167/iovs.13-13703 24722697 PMC4037937

[B54] Kissner A. Spoerl E. Jung R. Spekl K. Pillunat L. E. Raiskup F. (2010). Pharmacological modification of the epithelial permeability by benzalkonium chloride in UVA/Riboflavin corneal collagen cross-linking. Curr. Eye Res. 35, 715–721. 10.3109/02713683.2010.481068 20673048

[B55] Knox Cartwright N. E. Tyrer J. R. Marshall J. (2012). *In vitro* quantification of the stiffening effect of corneal cross-linking in the human cornea using radial shearing speckle pattern interferometry. J. Refract Surg. 28, 503–508. 10.3928/1081597x-20120613-01 22833877

[B56] Koc M. Uzel M. M. Koban Y. Durukan I. Tekin K. Ylmazbas P. (2016). Comparison of results of accelerated corneal cross-linking with hypo-osmolar riboflavin solution performed on corneas thicker and thinner than 400 μm. Cornea 35, 151–156. 10.1097/ICO.0000000000000709 26655487

[B57] Koppen C. Wouters K. Mathysen D. Rozema J. Tassignon M. J. (2012). Refractive and topographic results of benzalkonium chloride-assisted transepithelial crosslinking. J. Cataract. Refract Surg. 38, 1000–1005. 10.1016/j.jcrs.2012.01.024 22624899

[B58] Kopsachilis N. Tsaousis K. T. Tsinopoulos I. T. Kruse F. E. Welge-Luessen U. (2013). A novel mechanism of UV-A and riboflavin-mediated corneal cross-linking through induction of tissue transglutaminases. Cornea 32, 1034–1039. 10.1097/ICO.0b013e31828a760d 23594766

[B59] Labate C. De Santo M. P. Lombardo G. Lombardo M. (2015). Understanding of the viscoelastic response of the human corneal stroma induced by riboflavin/UV-a cross-linking at the nano level. PLoS One 10, e0122868. 10.1371/journal.pone.0122868 25830534 PMC4382164

[B60] Labate C. Lombardo M. Lombardo G. De Santo M. P. (2017). Biomechanical strengthening of the human cornea induced by nanoplatform-based transepithelial riboflavin/UV-A corneal cross-linking. Invest. Ophthalmol. Vis. Sci. 58, 179–184. 10.1167/iovs.16-20813 28114577

[B61] Leong Y. Y. Tong L. (2015). Barrier function in the ocular surface: from conventional paradigms to new opportunities. Ocul. Surf. 13, 103–109. 10.1016/j.jtos.2014.10.003 25881994

[B62] Li X. Li Y. Kim M. Trokel S. L. Turro N. J. Paik D. C. (2014). Aliphatic beta-nitroalcohols for therapeutic corneoscleral cross-linking: chemical stability studies using 1H-nmr spectroscopy. Photochem Photobiol. 90, 338–343. 10.1111/php.12165 23998198 PMC3939070

[B63] Li X. Li Y. Rao Y. Solomon M. R. Paik D. C. Turro N. J. (2013). Mechanistic and catalytic studies of beta-nitroalcohol crosslinking with polyamine. J. Appl. Polym. Sci. 128, 3696–3701. 10.1002/app.38604 24596431 PMC3939017

[B64] Lidich N. Garti-Levy S. Aserin A. Garti N. (2019). Potentiality of microemulsion systems in treatment of ophthalmic disorders: Keratoconus and dry eye syndrome - *in vivo* study. Colloids Surf. B Biointerfaces 173, 226–232. 10.1016/j.colsurfb.2018.09.063 30300828

[B65] Lidich N. Wachtel E. J. Aserin A. Garti N. (2016). Water-dilutable microemulsions for transepithelial ocular delivery of riboflavin phosphate. J. Colloid Interface Sci. 463, 342–348. 10.1016/j.jcis.2015.02.011 26614391

[B66] Loftsson T. Stefansson E. (2017). Cyclodextrins and topical drug delivery to the anterior and posterior segments of the eye. Int. J. Pharm. 531, 413–423. 10.1016/j.ijpharm.2017.04.010 28391041

[B67] Lombardo G. Micali N. L. Villari V. Leone N. Serrao S. Rusciano D. (2017). Assessment of stromal riboflavin concentration-depth profile in nanotechnology-based transepithelial corneal crosslinking. J. Cataract. Refract Surg. 43, 680–686. 10.1016/j.jcrs.2017.03.026 28602332

[B68] Lorenzo-Martin E. Gallego-Munoz P. Ibares-Frias L. Marcos S. Perez-Merino P. Fernandez I. (2018). Rose bengal and green light versus riboflavin-UVA cross-linking: Corneal wound repair response. Invest. Ophthalmol. Vis. Sci. 59, 4821–4830. 10.1167/iovs.18-24881 30347076

[B69] Ma J. Wang Y. Wei P. Jhanji V. (2018). Biomechanics and structure of the cornea: implications and association with corneal disorders. Surv. Ophthalmol. 63, 851–861. 10.1016/j.survophthal.2018.05.004 29857022

[B70] Malhotra C. Jain A. K. Gupta A. Ram J. Ramatchandirane B. Dhingra D. (2017). Demarcation line depth after contact lens-assisted corneal crosslinking for progressive keratoconus: Comparison of dextran-based and hydroxypropyl methylcellulose-based riboflavin solutions. J. Cataract. Refract Surg. 43, 1263–1270. 10.1016/j.jcrs.2017.07.032 29120712

[B71] Mehta J. Takaoka A. Zyablitskaya M. Nagasaki T. Paik D. C. (2020). Development of a topical tissue cross-linking solution using sodium hydroxymethylglycinate (SMG): viscosity effect. Biosci. Rep. 40. 10.1042/BSR20191941 PMC6954362 31860073

[B72] Morrison P. W. Connon C. J. Khutoryanskiy V. V. (2013). Cyclodextrin-mediated enhancement of riboflavin solubility and corneal permeability. Mol. Pharm. 10, 756–762. 10.1021/mp3005963 23294178

[B73] Morrison P. W. J. Porfiryeva N. N. Chahal S. Salakhov I. A. Lacourt C. Semina I. (2017). Crown ethers: Novel permeability enhancers for ocular drug delivery? Mol. Pharm. 14, 3528–3538. 10.1021/acs.molpharmaceut.7b00556 28825493

[B74] Morrison P. W. Khutoryanskiy V. V. (2014). Enhancement in corneal permeability of riboflavin using calcium sequestering compounds. Int. J. Pharm. 472, 56–64. 10.1016/j.ijpharm.2014.06.007 24928133 PMC4111866

[B75] Nassaralla B. A. Vieira D. M. Machado M. L. Figueiredo M. N. Nassaralla J. J. Jr. (2013). Corneal thickness changes during corneal collagen cross-linking with UV-A irradiation and hypo-osmolar riboflavin in thin corneas. Arq. Bras. Oftalmol. 76, 155–158. 10.1590/s0004-27492013000300005 23929075

[B76] Ostacolo C. Caruso C. Tronino D. Troisi S. Laneri S. Pacente L. (2013). Enhancement of corneal permeation of riboflavin-5'-phosphate through vitamin E TPGS: A promising approach in corneal trans-epithelial cross linking treatment. Int. J. Pharm. 440, 148–153. 10.1016/j.ijpharm.2012.09.051 23046664

[B77] Ozmen M. C. Hondur A. Yilmaz G. Bilgihan K. Hasanreisoglu B. (2014). A histological study of rabbit corneas after transepithelial corneal crosslinking using partial epithelial photoablation or ethanol treatment. Int. J. Ophthalmol. 7, 959–963. 10.3980/j.issn.2222-3959.2014.06.08 25540746 PMC4270988

[B78] Paik D. C. Solomon M. R. Wen Q. Turro N. J. Trokel S. L. (2010). Aliphatic beta-nitroalcohols for therapeutic corneoscleral cross-linking: chemical mechanisms and higher order nitroalcohols. Invest. Ophthalmol. Vis. Sci. 51, 836–843. 10.1167/iovs.09-3937 19797229 PMC2868449

[B79] Paik D. C. Wen Q. Braunstein R. E. Airiani S. Trokel S. L. (2009). Initial studies using aliphatic beta-nitro alcohols for therapeutic corneal cross-linking. Invest. Ophthalmol. Vis. Sci. 50, 1098–1105. 10.1167/iovs.08-2202 18836172 PMC2675911

[B80] Palazzo M. Vizzarri F. Ondruska L. Rinaldi M. Pacente L. Guerra G. (2020). Corneal UV protective effects of a topical antioxidant formulation: A pilot study on *in vivo* rabbits. Int. J. Mol. Sci. 21, 5426. 10.3390/ijms21155426 32751471 PMC7432813

[B81] Raiskup F. Pinelli R. Spoerl E. (2012). Riboflavin osmolar modification for transepithelial corneal cross-linking. Curr. Eye Res. 37, 234–238. 10.3109/02713683.2011.637656 22335811

[B82] Raiskup F. Theuring A. Pillunat L. E. Spoerl E. (2015). Corneal collagen crosslinking with riboflavin and ultraviolet-A light in progressive keratoconus: ten-year results. J. Cataract. Refract Surg. 41, 41–46. 10.1016/j.jcrs.2014.09.033 25532633

[B83] Rapuano P. B. Mathews P. M. Florakis G. J. Trokel S. L. Suh L. H. (2018). Corneal collagen crosslinking in patients treated with dextran versus isotonic hydroxypropyl methylcellulose (HPMC) riboflavin solution: A retrospective analysis. Eye Vis. (Lond) 5, 23. 10.1186/s40662-018-0116-z 30214908 PMC6130056

[B84] Rossi S. Orrico A. Santamaria C. Romano V. De Rosa L. Simonelli F. (2015). Standard versus trans-epithelial collagen cross-linking in keratoconus patients suitable for standard collagen cross-linking. Clin. Ophthalmol. 9, 503–509. 10.2147/OPTH.S73991 25834386 PMC4370945

[B85] Rubinfeld R. S. Gum G. G. Talamo J. H. Parsons E. C. (2021). The effect of sodium iodide on stromal loading, distribution and degradation of riboflavin in a rabbit model of transepithelial corneal crosslinking. Clin. Ophthalmol. 15, 1985–1994. 10.2147/OPTH.S300886 34007152 PMC8123948

[B86] Rubinfeld R. S. Stulting R. D. Gum G. G. Talamo J. H. (2018). Quantitative analysis of corneal stromal riboflavin concentration without epithelial removal. J. Cataract. Refract Surg. 44, 237–242. 10.1016/j.jcrs.2018.01.010 29526339

[B87] Salimi A. Gauvin M. Harissi-Dagher M. Racine L. Cohen M. Wallerstein A. (2022). Hypo-osmolar accelerated corneal crosslinking on resultant sub-400 μm topography-guided excimer regularized keratoconus corneas. J. Cataract. Refract Surg. 48, 1366–1374. 10.1097/j.jcrs.0000000000000993 35786809

[B88] Samaras K. O'brart D. P. Doutch J. Hayes S. Marshall J. Meek K. M. (2009). Effect of epithelial retention and removal on riboflavin absorption in porcine corneas. J. Refract Surg. 25, 771–775. 10.3928/1081597X-20090813-03 19772262

[B89] Santodomingo-Rubido J. Carracedo G. Suzaki A. Villa-Collar C. Vincent S. J. Wolffsohn J. S. (2022). Keratoconus: An updated review. Cont. Lens Anterior Eye 45, 101559. 10.1016/j.clae.2021.101559 34991971

[B90] Silvestre J. Chen S. Zheng Z. Vega A. Chen T. Rodriguez-Reinoso F. (2022). Carbon nanostructures for ocular tissue reinforcement. Transl. Vis. Sci. Technol. 11, 1. 10.1167/tvst.11.9.1 PMC9440608 36048013

[B91] Singh T. Taneja M. Murthy S. Vaddavalli P. K. (2020). Evaluation of safety and efficacy of different protocols of collagen cross linking for keratoconus. Rom. J. Ophthalmol. 64, 158–167. 10.22336/rjo.2020.28 32685782 PMC7339692

[B92] Song W. Cheng Y. Yan X. Yang S. (2021). Long-term study of corneal stroma and endothelium on structure and cells after genipin treatment of rabbit corneas. Transl. Vis. Sci. Technol. 10, 9. 10.1167/tvst.10.5.9 PMC8447043 34529024

[B93] Song W. Tang Y. Qiao J. Li H. Rong B. Yang S. (2017). The comparative safety of genipin versus UVA-riboflavin crosslinking of rabbit corneas. Mol. Vis. 23, 504–513. 28761323 PMC5524432

[B94] Song W. Tang Y. Qiao J. Li H. Rong B. Yang S. (2019). The short-term safety evaluation of corneal crosslinking agent-genipin. Ophthalmic Res. 62, 141–149. 10.1159/000499571 31112970

[B95] Spadea L. Mencucci R. (2012). Transepithelial corneal collagen cross-linking in ultrathin keratoconic corneas. Clin. Ophthalmol. 6, 1785–1792. 10.2147/OPTH.S37335 23152657 PMC3497455

[B96] Spoerl E. Huhle M. Seiler T. (1998). Induction of cross-links in corneal tissue. Exp. Eye Res. 66, 97–103. 10.1006/exer.1997.0410 9533835

[B97] Stojanovic A. Chen X. Jin N. Zhang T. Stojanovic F. Raeder S. (2012). Safety and efficacy of epithelium-on corneal collagen cross-linking using a multifactorial approach to achieve proper stromal riboflavin saturation. J. Ophthalmol. 2012, 498435. 10.1155/2012/498435 22900147 PMC3413959

[B98] Tang Y. Song W. Qiao J. Rong B. Wu Y. Yan X. (2019). A study of corneal structure and biomechanical properties after collagen crosslinking with genipin in rabbit corneas. Mol. Vis. 25, 574–582. 31673223 PMC6798704

[B99] Thangavel N. Jayakumar I. Ravichandran M. Vaidyanathan Ganesan V. Nair B. U. (2019). Photocrosslinking of collagen using Ru(II)-polypyridyl complex functionalized gold nanoparticles. Spectrochim. Acta A Mol. Biomol. Spectrosc. 215, 196–202. 10.1016/j.saa.2019.02.098 30826578

[B100] Thorsrud A. Hagem A. M. Sandvik G. F. Drolsum L. (2019). Superior outcome of corneal collagen cross-linking using riboflavin with methylcellulose than riboflavin with dextran as the main supplement. Acta Ophthalmol. 97, 415–421. 10.1111/aos.13928 30284383

[B101] Torricelli A. A. Ford M. R. Singh V. Santhiago M. R. Dupps W. J. Jr. Wilson S. E. (2014). BAC-EDTA transepithelial riboflavin-UVA crosslinking has greater biomechanical stiffening effect than standard epithelium-off in rabbit corneas. Exp. Eye Res. 125, 114–117. 10.1016/j.exer.2014.06.001 24929203 PMC4128899

[B102] Tsao S. Yao M. Tsao H. Henry F. P. Zhao Y. Kochevar J. J. (2012). Light-activated tissue bonding for excisional wound closure: A split-lesion clinical trial. Br. J. Dermatol 166, 555–563. 10.1111/j.1365-2133.2011.10710.x 22032650

[B103] Ustundag Okur N. Caglar E. S. Siafaka P. I. (2020). Novel ocular drug delivery systems: An update on microemulsions. J. Ocul. Pharmacol. Ther. 36, 342–354. 10.1089/jop.2019.0135 32255728

[B104] Vaidya N. S. Daneshmand A. Epstein R. J. Majmudar P. A. Belin M. W. Parsons E. C. (2022). Pachymetric assessment after EpiSmart^®^ epithelium-on cross-linking for keratoconus and post-surgical ectasia. Clin. Ophthalmol. 16, 1829–1835. 10.2147/OPTH.S359710 35702687 PMC9188777

[B105] Verter E. E. Gisel T. E. Yang P. Johnson A. J. Redmond R. W. Kochevar I. E. (2011). Light-initiated bonding of amniotic membrane to cornea. Invest. Ophthalmol. Vis. Sci. 52, 9470–9477. 10.1167/iovs.11-7248 22058339

[B106] Vohra V. Tuteja S. Gurnani B. Chawla H. (2023). “Collagen cross linking for keratoconus,” in StatPearls (Treasure Island, FL: StatPearls Publishing). 32965942

[B107] Wang T. Zhu L. Zhu J. Peng Y. Shen N. Yu Y. (2018). Subacute effects of rose Bengal/Green light cross linking on rabbit thin corneal stability and safety. Lasers Surg. Med. 50, 324–332. 10.1002/lsm.22762 29095506

[B108] Wang X. Majumdar S. Ma G. Sohn J. Yiu S. C. Stark W. (2017). Chondroitin sulfate-based biocompatible crosslinker restores corneal mechanics and collagen alignment. Invest. Ophthalmol. Vis. Sci. 58, 3887–3895. 10.1167/iovs.16-21292 28763562

[B109] Wang Y. Wang Z. Dong Y. (2023). Collagen-based biomaterials for tissue engineering. ACS Biomater. Sci. Eng. 9, 1132–1150. 10.1021/acsbiomaterials.2c00730 36800415

[B110] Wen Q. Trokel S. L. Kim M. Paik D. C. (2013). Aliphatic beta-nitroalcohols for therapeutic corneoscleral cross-linking: corneal permeability considerations. Cornea 32, 179–184. 10.1097/ICO.0b013e31825646de 22868628 PMC3493702

[B111] Wertheimer C. M. Elhardt C. Kaminsky S. M. Pham L. Pei Q. Mendes B. (2019). Enhancing rose bengal-photosensitized protein crosslinking in the cornea. Invest. Ophthalmol. Vis. Sci. 60, 1845–1852. 10.1167/iovs.19-26604 31042790

[B112] Wollensak G. Iomdina E. (2009). Biomechanical and histological changes after corneal crosslinking with and without epithelial debridement. J. Cataract. Refract Surg. 35, 540–546. 10.1016/j.jcrs.2008.11.036 19251149

[B113] Wollensak G. Spoerl E. Seiler T. (2003). Riboflavin/ultraviolet-a-induced collagen crosslinking for the treatment of keratoconus. Am. J. Ophthalmol. 135, 620–627. 10.1016/s0002-9394(02)02220-1 12719068

[B114] Wollensak G. Sporl E. (2019). Biomechanical efficacy of corneal cross-linking using hypoosmolar riboflavin solution. Eur. J. Ophthalmol. 29, 474–481. 10.1177/1120672118801130 30255714

[B115] Wu J. Wang J. Wang L. Huang Y. (2022). Topical retinoic acid induces corneal strengthening by upregulating transglutaminase 2 in murine cornea. Exp. Eye Res. 214, 108850. 10.1016/j.exer.2021.108850 34861212

[B116] Wu Y. Song W. Tang Y. Elsheikh A. Shao Y. Yan X. (2019). Efficacy and safety of transglutaminase-induced corneal stiffening in rabbits. Transl. Vis. Sci. Technol. 8, 27. 10.1167/tvst.8.6.27 PMC6908136 31853423

[B117] Wu Y. Song W. Tang Y. Yan X. (2020). Biomechanical changes after *in vivo* enzyme-induced corneal crosslinking in rabbits. Ophthalmic Res. 63, 501–506. 10.1159/000505629 31884496

[B118] Xue A. Zheng L. Tan G. Wu S. Wu Y. Cheng L. (2018). Genipin-crosslinked donor sclera for posterior scleral contraction/reinforcement to fight progressive myopia. Invest. Ophthalmol. Vis. Sci. 59, 3564–3573. 10.1167/iovs.17-23707 30025077

[B119] Yang J. Yang Y. W. (2020). Metal-organic frameworks for biomedical applications. Small 16, e1906846. 10.1002/smll.201906846 32026590

[B120] Yang M. Xu W. Chen Z. Chen M. Zhang X. He H. (2022). Engineering hibiscus-like riboflavin/ZIF-8 microsphere composites to enhance transepithelial corneal cross-linking. Adv. Mater 34, e2109865. 10.1002/adma.202109865 35316534

[B121] Yurttaser Ocak S. Mangan M. S. (2021). Endothelial cell loss after accelerated corneal crosslinking using pachymetry-guided hypo-osmolar riboflavin dosing in thin keratoconic corneas. J. Cataract. Refract Surg. 47, 1530–1534. 10.1097/j.jcrs.0000000000000686 34074991

[B122] Zaheer N. Khan W. A. Khan S. Khan M. A. M. (2018). Comparison of changes in central corneal thickness during corneal collagen cross-linking, using isotonic riboflavin solutions with and without dextran, in the treatment of progressive keratoconus. Cornea 37, 340–346. 10.1097/ICO.0000000000001496 29283924

[B123] Zhu H. Alt C. Webb R. H. Melki S. Kochevar I. E. (2016). Corneal crosslinking with rose bengal and green light: Efficacy and safety evaluation. Cornea 35, 1234–1241. 10.1097/ICO.0000000000000916 27362877

